# Species traits as predictors for intrinsic sensitivity of aquatic invertebrates to the insecticide chlorpyrifos

**DOI:** 10.1007/s10646-012-0962-8

**Published:** 2012-06-19

**Authors:** Mascha N. Rubach, Donald J. Baird, Marie-Claire Boerwinkel, Stephen J. Maund, Ivo Roessink, Paul J. Van den Brink

**Affiliations:** 1 Department of Aquatic Ecology and Water Quality Management, Wageningen University, P.O. Box 47, 6700 AA Wageningen, The Netherlands; 2 Syngenta Crop Protection AG, 4002 Basel, Switzerland; 3 Environment Canada@Canadian Rivers Institute, Department of Biology, University of New Brunswick, Fredericton, NB Canada; 4 Alterra, Wageningen University and Research Centre, P.O. Box 47, 6700 AA Wageningen, The Netherlands

**Keywords:** Toxicokinetics–toxicodynamics, Sensitivity, Species traits, Pesticides, Prediction

## Abstract

Ecological risk assessment (ERA) has followed a taxonomy-based approach, making the assumption that related species will show similar sensitivity to toxicants, and using safety factors or species sensitivity distributions to extrapolate from tested to untested species. In ecology it has become apparent that taxonomic approaches may have limitations for the description and understanding of species assemblages in nature. Therefore it has been proposed that the inclusion of species traits in ERA could provide a useful and alternative description of the systems under investigation. At the same time, there is a growing recognition that the use of mechanistic approaches in ERA, including conceptual and quantitative models, may improve predictive and extrapolative power. Purposefully linking traits with mechanistic effect models could add value to taxonomy-based ERA by improving our understanding of how structural and functional system facets may facilitate inter-species extrapolation. Here, we explore whether and in what ways traits can be linked purposefully to mechanistic effect models to predict intrinsic sensitivity using available data on the acute sensitivity and toxicokinetics of a range of freshwater arthropods exposed to chlorpyrifos. The results of a quantitative linking of seven different endpoints and twelve traits demonstrate that while quantitative links between traits and/or trait combinations and process based (toxicokinetic) model parameters can be established, the use of simple traits to predict classical sensitivity endpoints yields little insight. Remarkably, neither of the standard sensitivity values, i.e. the LC_50_ or EC_50_, showed a strong correlation with traits. Future research in this area should include a quantitative linking of toxicodynamic parameter estimations and physiological traits, and requires further consideration of how mechanistic trait-process/parameter links can be used for prediction of intrinsic sensitivity across species for different substances in ERA.

**Electronic supplementary material:**

The online version of this article (doi:10.1007/s10646-012-0962-8) contains supplementary material, which is available to authorized users.

## Introduction

Traits, or specific characteristics, like gill or air breathing have been used in ecology for almost 100 years and have increased our understanding of the persistence of species assemblages, or ecological communities, in relation to their habitat (e.g. Thienemann 1918; Poff et al. 2006). This has led to a systematic application of traits in biomonitoring and retrospective environmental and conservation risk assessment (Culp et al. 2011; Van den Brink et al. 2011a). In ecotoxicology, traits have also been used in some quantitative structure activity relationship (QSAR) models (Escher and Hermens 2004), the SPEAR index (Liess and Von Der Ohe 2005), in corrections of certain risk indicators for specific traits (e.g. lipid correction of bioconcentration factors), effect analysis and modelling using life table and life history traits (Arnot and Gobas 2004; Preuss et al. 2009) and in test battery optimization (Ducrot et al. 2005). However, it is rare that more than one or two traits are incorporated into these approaches, and the acquisition and use of traits has been made haphazardly, and thus the potential of traits for prospective environmental risk assessment remains to be fully explored (Baird et al. 2008). Prospective risk assessment, which is based on probabilistic assumptions and empirical data linked to mechanistic knowledge, could benefit from traits-based approaches, particularly when combined with predictive models (Rubach et al. 2011a). The application of effect models for ecotoxicology and ecological risk assessment (ERA), including toxicokinetic (TK)–toxicodynamic (TD) models and individual based (population) models (IBMs), has been and is being explored by various authors (e.g. Galic et al. 2010; Schmolke et al. 2010) and workshops (e.g. Thorbek et al. 2010; Van den Brink et al. 2011b). The combined potential of effect models for ERA and traits-based approaches could be significant, as it would offer a means to facilitate interspecies extrapolation, one of the major challenges facing the future refinement of ERA (Rubach et al. 2011a). The exploration of such an approach will be highly dependent on the establishment of mechanistic links between the relevant traits and processes and the availability of suitable data (Rubach et al. 2011a). In order to explore the intrinsic sensitivity of freshwater arthropods a traits-based effect model was developed and appropriate calibration data for a model compound (chlorpyrifos) were previously collected by Rubach et al. (2010a, 2011b). This model compound was chosen, because acetylcholinesterase inhibition is a very specific mode of action and herewith related to the idea to link traits to process based parameters of toxicokinetics and toxicodynamics. Furthermore, the aquatic ecotoxicology of chlorpyrifos has been extensively studied at different levels of organization (molecular to ecosystem) and so was a suitable choice for the research not only because of the good understanding of its’ specific mode of action, but also the availability of a range of data to help guiding the research questions. A disadvantage of choosing chlorpyrifos is that it needs to oxidize to chlorpyrifos-oxon in order to be biologically active and exhibit efficacy. The rate of transformation to this biologically active metabolite would therefore be an indispensible parameter to be linked to physiological traits.

Intrinsic sensitivity is the integrated organism-level result of several internal processes and threshold values, i.e., uptake, biotransformation and elimination (summarized as toxicokinetics, TK) and damage/hazard, internal recovery and thresholds (summarized as toxicodynamics, TD) (Ashauer et al. 2007). The aforementioned TKTD models link external exposure and survival effects by describing dynamically the processes of TK and TD. Data mining of existing toxicity and trait data in order to establish empirical links between traits and sensitivity led to the conclusion that existing data are not suitable for calibration of traits-based effect models and further experimental work on the processes of toxicity and also a more purposeful collection of trait data should be explored (Rubach et al. 2010b). Rubach et al. (2011b) characterized the variation in sensitivities of a dataset of toxicity values for chlorpyrifos in freshwater arthropods to chlorpyrifos in the form of 24–96 h L(E)C_*X*_ values (*x*% lethal or effective concentrations) and also addressed species differences in response dynamics at lethal and sub-lethal levels. Rubach et al. (2010a) used this information to perform ^14^C-labelled bioconcentration experiments under sub-lethal exposure to chlorpyrifos with the same test species in order to parameterize the processes of uptake and elimination (TK) by means of a one-compartment, first order kinetic model for a range of freshwater arthropod species, which varied in their sensitivity and trait composition. As total radioactivity was not characterized in these studies and the ^14^C label was located at the ethyl substituent of chlorpyrifos, no quantification of transformation rates to the biologically active oxon metabolite are available. For a detailed discussion of this complication please refer to Rubach et al. (2010a).

These two studies delivered a comprehensive dataset on classical sensitivity endpoints and toxicokinetic (process based) parameters, which are linked here to a collection of traits in order to explore the conceptual model proposed by Rubach et al. (2011a) in more detail, to establish links between the toxicokinetic processes (uptake and elimination) and relevant traits that may be useful as predictors in a bioconcentration model. To achieve this end, we first populated a traits database composed of an a priori selection of traits. As a second step, we linked classical sensitivity (L(E)C_50_) and toxicokinetic (uptake and elimination rate constants, bioconcentration factors) parameters to these traits by means of principal component analysis (PCA) and linear regression models. These quantitative links are discussed both from a trait and from a process-based perspective. Finally these results are used to outline future predictive traits-based research needs and to discuss the implications of traits-based approaches for environmental risk assessment in the context of the present work.

## Materials and methods

### Trait selection

Empirical traits-based approaches face the challenge of how to make an a priori selection of traits to be included or excluded from any analysis. Definition and selection of traits are both somewhat subjective processes, and the proliferation of generally incompatible traits databases in the literature reflects the scope of this challenge (Baird et al. 2011). Here, in order to provide a rationale for the a priori selection of traits, we developed the following criteria:

(i) Traits for which mechanistic hypotheses linked to the processes of interest exist and/or previous empirical evidence for these has been found;
(ii) Traits which are possible to measure, and which could have a plausible relationship to sensitivity, although they might not be the best descriptor for these underlying physiological characteristics;
(iii) Traits, which fulfil (i) and (ii) and additionally relate to the experimental design of the study.

A priori trait selection was subsequently made, based on information gained and hypotheses generated in previous studies regarding traits and sensitivity, especially the studies of Baird and Van den Brink (2007) and Rubach et al. (2010b). Following this approach, traits were evaluated in terms of our ability to quantify them using existing literature and databases or by direct measurement from the same populations and/or sub-samples of the specimen used by Rubach et al. (2010a, 2011b). The selected traits and their modalities are listed in Table 1, including their abbreviated names, their coding type and the literature sources used for their quantification. In the Supplementary Material A and D, the traits used and their modalities are introduced, along with how the information on traits was obtained i.e. from the literature or through experimental quantification. Supplementary Material A also presents and discusses the trait by species matrix, which resulted from the analysis.

**Table 1:** Populated trait(s)/groups, their quantifications/modalities, short-form names and origin

Short-form names	Trait (group)	Quantification/modality	Unit	Type of variable	References^a^
Biovol	Size related	Biovolume	mm^3^	Metric	This study
SurfArea		Surface area (without gills)	mm^2^	Metric	This study
AVratio		Surface area/volume ratio	mm^−1^	Metric	This study
Length		Body length	mm	Metric	This study
DryMass		Dry mass	mg/individual	Metric	This study
WatCont	Water content	Water content	%	Metric	This study/Rubach et al. (2010a)
ExoTh	Thickness of exoskeleton	Thickness of exoskeleton	mm	Metric	This study
LipFW	Lipid content	% Lipid of wet weight	% wet weight	Metric	Rubach et al. (2010a)
LipDW		% Lipid of dry weight	% dry weight	Metric	Rubach et al. (2010a))
LipTot		Total lipid content	mg/individual	Metric	Rubach et al. (2010a)
ResConf	Respiratory regulation	Conformer	–	Binary	Welch (1922), Wingfield (1939), Mill and Hughes (1966), Wichard (1978), Babula (1979), Steele and Steele (1991), Taylor and Taylor (1992), Maltby (1995), Ueno et al. (1997), Pirow et al. (1999), Freire et al. (2008), Merritt et al. (2008)
ResInt		Intermediate	–	Binary	
ResReg		Regulator	–	Binary	
SOatm	Source of oxygen	Atmospheric oxygen	–	Binary	
SOdiss		Dissolved oxygen	–	Binary	
ResMocut	Mode of respiration^b^	Cutaneous	–	Binary	
ResMosip		Siphon	–	Binary	
ResMoCoG		Compressible gill	–	Binary	
ResMoExG		External gills	–	Binary	
ResMoInG		Internal gills	–	Binary	
ResMoPig		Respiratory pigments	–	Binary	
TroDetr	Trophic relation	Detritivore	–	Binary	Brown (1960), Williams (1962a, b), Hickin (1967), McShaffrey and McCafferty (1990), Jalihal et al. (1994), Schuh and Slater (1995), Gupta and Stewart (2000), Yee et al. (2004), Locklin et al. (2006), Merritt et al. (2008)
TroHerb		Herbivore	–	Binary	
TroCarn		Carnivore	–	Binary	
TroOmni		Omnivore	–	Binary	
SclPoor	Degree of sclerotization	Poor (<10 %)	–	Binary	This study; Poff et al. (2006) (trait armouring); Merritt et al. (2008)
SclGood		Good (10–90 %)	–	Binary	
SclComp		Complete (>90 %, carapace)	–	Binary	
BauBox	‘Bauplan’—shape of the organism	(Rectangular) box shapes	–	Binary	This study
BauCyl		Cylindroid	–	Binary	
BauSphe		Spheres and ellipsoids	–	Binary	
BauCone		Cones and half cones	–	Binary	
Ladult	Life stage	Adult	–	Binary	This study
Llarny		Larva/nymph	–	Binary	
Ljuv		Juvenile	–	Binary	
PhylRES^c^	Phylogeny	Rank species (lowest rank = oldest)	–	Ordinal	This study, based on Maddison and Maddison (1996)
PhylEQ^c^		Rank taxon (lowest rank = oldest)	–	Ordinal	

^a^References are added as Supplementary Material D

^b^Species tested did not account for the modes of respiration ‘plant breather’ and ‘incompressible gill’, which are therefore not listed as modalities here

^c^Based on phylogenetic tree of ‘Tree of life web project’ (Maddison and Maddison 1996), retrieved on 01.09.2009: counted nodes of lowest taxonomic resolution possible (family/(sub)/(infra)-order) back to common ancestor for arthropods (see text)

### Linking traits to sensitivity

In order to identify biological factors which cause differences in toxicokinetics and sensitivity across species and also explore how these relationships can be expressed quantitatively, the trait database provided in Supplementary Material A was quantitatively linked to seven different endpoints, which were characterized for the 17 species described in Rubach et al. (2010a, 2011b). Two of the seven different endpoints are routine endpoints used in ecotoxicology, namely the median lethal concentration in 48 h constant exposure (LC_50_ 48 h), and the median effective concentration for immobility in 48 h constant exposure (EC_50_ 48 h), both originating from Rubach et al. (2011b). Furthermore, as toxicokinetic parameters, the uptake rate constant (*k*_in_) corrected for fresh weight, the uptake rate constant, but uncorrected for fresh weight *(k*_in, uncorr_), the elimination rate constant (*k*_out_), the bioconcentration factor (BCF) corrected for fresh weight (BCF_ww_) and the BCF corrected for lipid content (BCF_lipid_) were all taken from Rubach et al. (2010a). These seven endpoints and the values used are displayed in Table 2.

**Table 2:** The five toxicokinetic and two sensitivity endpoints used to link traits and sensitivity

Species	Endpoints						
	*k*_in_^a^ (L kg_ww_^−1^ day^−1^)	*k*_in, uncorr_^a^ (L day^−1^)	*k*_out_^a^ (day^−1^)	BCF_ww_^a^ (L kg_ww_^−1^)	BCF_lipid_^a^ (L kg_lipid_^−1^)	48 h LC_50_^b^ (μg L^−1^)	48 h EC_50_^b^ (μg L^−1^)
*Anax imperator*	21.2	0.02993	0.212	100	4021	3.29	3.134
*Asellus aquaticus*	596	0.00683	0.185	3242	382956	n.c.^c^	6.159
*Chaoborus obscuripes*	318	0.00555	0.131	2428	234140	1.13	0.438
*Cloeon dipterum*	349	0.00268	0.196	1782	24699	0.81	0.763
*Culex pipiens*	328	0.00112	0.024	13930	1999644	0.2	n.p.^f^
*Daphnia magna*	295	0.00398	0.546	541	57437	27.43	0.484
*Gammarus pulex* (AD)	812	0.01554	0.398	2039	149919	0.43	0.379
*Gammarus pulex* (JU)	1110	0.00520	0.36	3083	627029	n.p.^d^	n.p.^d^
*Molanna angustata*	579	0.01362	0.109	5331	181901	z.m.^e^	1.857
*Neocaridinia denticulata*	617	0.02459	0.478	1291	103599	660.1	327.2
*Notonecta maculata*	61.9	0.00823	0.152	407	10679	23.938	9.071
*Parapoynx stratiotata*	275	0.00930	0.171	1601	35458	29.41	2.94
*Plea minutissima*	88.2	0.00043	0.135	654	8592	5.94	2.645
*Procambarus spec.* (AD)	24.2	0.06762	0.086	280	14220	34.81	20.727
*Procambarus spec.* (JU)	199	0.00516	0.154	1295	111332	2.75	1.702
*Ranatra linearis*	42.1	0.00617	0.107	392	40891	11.97	11.97
*Sialis lutaria*	203	0.00806	0.021	9625	500412	z.m.^e^	1.548

^a^Taken from Rubach et al. (2010a), *k*_in_ and *k*_out_ based on the Markov-Chain-Monte–Carlo estimates

^b^Taken from Rubach et al. (2011b)

^c^Not computed, for analysis the 72 h LC_50_ of 7.639 μg L^−1^ was used

^d^Not performed, for analysis the values of *G. pulex* adults were used

^e^Zero mortality observed, for analysis value of 10000 was used

^f^Not performed, the LC_50_ was used

A quantitative, two-step approach was chosen to link the endpoints to traits, with both steps assuming a linear relationship. In step one, a principal components analysis (PCA) was performed using the species by trait matrix as species data and the species by endpoints matrix as passive explanatory variables. This PCA was performed to explore how traits grouped with each other on basis of our species selection and how this variation related to the variation in the endpoints measured. Before the PCA was performed, the metric traits were square root transformed and the endpoint data were log transformed. The PCA was performed in Canoco for Windows Version 4.5 (Ter Braak and Smilauer 2002).

In step two, single and multiple linear regressions were performed with the same data with the following three objectives: (i) to evaluate the explanatory potential of single traits and to identity their relevance for each endpoint (single regressions); (ii) to extract combinations of traits with explanatory potential for each endpoint and to evaluate their relevance for the processes of toxicity (multiple regressions); (iii) to identify the best endpoint(s) and the appropriate level of mechanistic detail needed to make quantitative links between traits and the endpoints.

In order to identify single traits and combinations of traits as factors with high explanatory and therefore predictive potential for intrinsic sensitivity, a linear regression selection method was performed using the RSEARCH procedure in GenStat release 12.1 (Payne 2007). Subsequently for all traits, simple single linear regressions were performed with all trait quantifications/modalities and each of the seven endpoints separately. Thereafter, from each of the 12 traits, the one quantification or modality which explained the largest variance in the respective endpoint was selected for the forward multiple regressions, irrespective of significance. Also, trait modalities were omitted from an analysis if they were strongly correlated (e.g. although frequently significant in the single linear regressions, LipTot was removed from all subset analysis because correlation analysis indicated strong correlations with all size-related variables except the surface-area-volume-ratio (AVratio)). The selected trait quantifications and modalities (always a set of 12 traits) were then used as candidate regression models (all possible combinations) that were then analysed separately using a multiple regression. The term ‘significant’ is used in reference to an accepted 5 % probability for type I errors (*p* ≤ 0.05), while ‘moderately significant’ refers to 10 % accepted error probability (0.05 < *p* ≤ 0.1). The latter significance levels are included due to the anticipated high levels background variation in the combined datasets.

## Results

### Principal component analysis

**Fig. 1:**
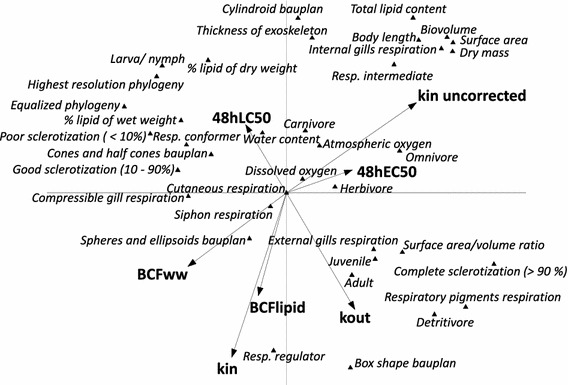
PCA biplot showing the variation in traits of different species and their relationship with several sensitivity endpoints. The first axis displays 21 % of the total variation in traits between the taxa and 22 % of the variation in sensitivity parameters, while the second axis another 19 % of the variation in traits and 30 % of the variation in sensitivity parameters. Abbreviations of the traits are explained in Table 1, those of the sensitivity endpoints in Table 2

The results are presented in Fig. 1 as a PCA biplot with an overlay of the explanatory variables. The overlay of the endpoints shows that the uncorrected *k*_in_ was positively correlated with the size related measures (with the exception of the surface-area-volume-ratio (AVratio), which is not correlated at all) in contrast to the fresh-weight-corrected *k*_in, uncorr_, which is negatively correlated. Similarly, clear correlations with uptake are indicated also for total lipid content (LipTot), exoskeleton thickness (ExoTh) and being an intermediate respiratory regulator (ResInt). The endpoints for bioconcentration are both highly correlated with the corrected *k*_in_ and therefore related in the same pattern to the same traits. For the toxicokinetic endpoint *k*_out_, the PCA suggests that adult (Ladult) and juvenile (Ljuv) stages have better elimination abilities than larval/nymphal stages (Larny). Also the traits ‘complete sclerotization’ (SclComp), ‘being a detritivore’ (TroDetr), or ‘being equipped with respiratory pigments’ (ResMoPig) appear to be associated with high elimination rate constants. In turn, many traits are negatively correlated with *k*_out_, including ‘lipid content’ (Lip), ‘poor sclerotization’ (SclPoor) and ‘high relative rank in phylogenetic position (late separation from arthropod lineage)’ (PhylRES). Remarkably, in terms of their correlation with traits, neither of the standard toxicity values, the LC_50_ or EC_50_ showed a strong correlation in this analysis.

### Linear regression analysis

In order to give an overview about the relevance of each trait quantification/modality investigated, the significance and coefficient of determination (adjusted *R* ^2^) of all single linear regressions are shown in Table 3 and Supplemental Material B. In both tables, boldface is used to indicate which trait variables were selected for the forward multiple regressions for which the results are displayed in Supplementary Material C.

**Table 3:** Results of single linear regressions (each sensitivity endpoint regressed against each trait modality/quantification)

Trait (modality)	Adj. *R* ^2^													
	*k*_in_ (L kg_ww_^−1^ day^−1^)		*k*_in, uncorr_ (L day^−1^)		*k*_out_ (day^−1^)		BCF_ww_ (L kg_ww_^−1^)		BCF_lipid_ (L kg_lipid_^−1^)		48 h LC_50_ [μg·L^−1^]		48 h EC_50_ [μg·L^−1^]	
Biovolume	1 (−)	0.471**	1 (+)	0.379**			1 (−)	0.316**	2 (−)	0.182**				
Surface Area	**1** (−)	0.56**	1 (+)	0.326**			**1** (−)	0.391**	**2** (−)	0.2**				
AVratio														
Length	1 (−)	0.474**	**1** (+)	0.545**			1 (−)	0.298**	2 (−)	0.143*			**1** (+)	0.191**
DryMass	1 (−)	0.384**	1 (+)	0.338**			1 (−)	0.213**	2 (−)	0.125*				
WatCont														
ExoTh			**1** (+)	0.173*	**3** (−)	0.197**					**1** (+)	0.329**		
LipFW									**1** (−)	0.37**				
LipDW									1 (−)	0.156*				
LipTot^a^	2 (−)	0.558**	2 (+)	0.397**			2 (−)	0.392**	1 (−)	0.31**				
ResConf														
ResInt	**3** (−)	0.14*												
ResReg														
SOatm	**1** (−)	0.278**												
SOdiss					**3** (+)	0.157*								
ResMocut	NP	NP	NP	NP	NP	NP	NP	NP	NP	NP	NP	NP	NP	NP
ResMosip														
ResMoCoG			1 (−)	0.116*					**1** (−)	0.166*				
ResMoExG	1 (+)	0.237**												
ResMoInG	**3** (−)	0.304**	**3** (+)	0.158*			**3** (−)	0.231**						
ResMoPig					**3** (+)	0.252**								
TroDetr	**3** (+)	0.129*												
TroHerb														
TroCarn														
TroOmni														
SclPoor							**4** (−)	0.172*						
SclGood														
SclComp			**4** (+)	0.16*	**3** (+)	0.244**	**3** (−)	0.128*						
BauBox	**3** (+)	0.128*			**3** (+)	0.145*								
BauCyl														
BauSphe			**3** (−)	0.232**										
BauCone											**3** (+)	0.12*		
Ladult							**2** (+)	0.135*					**2** (+)	0.206**
Llarny					**3** (−)	0.186**								
Ljuv														
PhylRES					**3** (−)	0.194**								
PhylEQ					**3** (−)	0.295**								

Sign (±) and interpretation (1, 2, 3, 4) of significant relationship between single traits and sensitivity. A “1” denotes an expected, known relationship, a “2” a size artefact, a “3” an interesting, logical relationship, while a “4” denotes an illogical relationship. The p value reports the F statistic, notation for significance: * = *p* ≤ 0.1 and ** = *p* ≤ 0.05. The adj. *R* ^2^ = adjusted coefficient of determination, percent variance accounted for by trait combination. *NC* not calculated, because residual variance exceeded variance of response variable. *NP* not performed, because all species were quantified as cutaneous breathers. Boldface indicates the variable/modality selected for the forward multiple linear regression analyses

^a^Total lipid content was never selected for the forward multiple linear regressions in order to avoid artefact results in relation to body size

## Single regressions—single traits

In order to facilitate the interpretation of single trait-endpoint relationships, Table 3 only lists the trait variables that were significant in the single regressions for each endpoint separately, also indicating the direction (positive or negative) of the relationships as well as their relevance as a result of their discussion (e.g. if a result is a size artefact). The complete results of the single regressions can be found in Supplemental Material B.

Size-related traits (except AVratio) and LipTot showed highly significant negative relationships with uptake and bioconcentration of chlorpyrifos in freshwater arthropods. The body length (Length) of a species was also significantly correlated with its 48 h EC_50_. The trait ‘water content’ (WatCont) was not significantly related to any of the endpoints, while exoskeleton thickness (ExoTh) was moderately significantly related to *k*_in, uncorr_, 48 h LC_50_ and *k*_out_. Of the traits describing lipid content, LipTot was significantly related to uptake and BCF endpoints. The fresh and dry weight corrected lipid contents (LipFW, LipDW) were (moderately) significantly negatively related to the BCF_lipid_ (Table 3).

Abilities to regulate respiration (Res) did not show strong relationships with any of the endpoints. Only having intermediate regulatory capabilities (ResInt) was found to decrease uptake moderately significantly (Table 3 and Supplementary Material B). Among the single traits addressing respiration, the strongest explanatory power for *k*_in_ was observed for sourcing atmospheric oxygen (SOatm), for a mode of respiration using an external gill (ResMoExG) and internal gill (ResMoInG), while compressible gills ResMoCoG and internal gills ResMoInG were correlated with *k*_in, uncorr_. The analysis indicated that species using dissolved oxygen for respiration (SOdiss) were moderately significantly positively correlated with *k*_out_ (Table 3 and Supplementary Material B). Siphon (ResMosip) and pigments (ResMoPig) respiration were also (moderately) significantly correlated with *k*_out_. Respiration via internal gills (ResMoInG) also showed a negative relationship with BCF_ww_, while ResMoCoG was significantly correlated with BCF_lipid_. Detritivore food preference (TroDetr) was found to be the only trophic trait significantly related to the endpoints analysed, namely to increased uptake (Table 3 and Supplementary Material B).

The relationships calculated for traits related to the degree of sclerotization showed that complete sclerotization is moderately significant, positively correlated with uncorrected uptake (*k*_in, uncorr_) and negatively correlated with elimination. Both poor and complete sclerotization were moderately significantly correlated with BCF_ww_ (Table 3 and Supplementary Material B).

The trait bauplan (Bau) was adapted from the geometrical bodies used for the quantification of the AVratio, but these variables were only weakly correlated (correlation coefficients between 0.106 and 0.309). The box shaped modality was moderately significantly related to both enhanced uptake and elimination, while the cone shape was moderately significantly positively correlated with higher 48 h LC_50_ values, and the sphere-shaped body was correlated significantly negatively with *k*_in, uncorr_ (Table 3 and Supplementary Material B).

Adult life stage (Ladult) was moderately significantly correlated with *BCFww* and significantly correlated with the 48 h EC_50_, while larval/nymph life stage (Larny) was moderately significantly correlated with *k*_out_ (Table 3). The trait modalities accounting for phylogeny (PhyRES and PhyEQ) correlated significantly with *k*_out_ (Table 3).

### Multiple regressions—combinations of traits

**Fig. 2:**
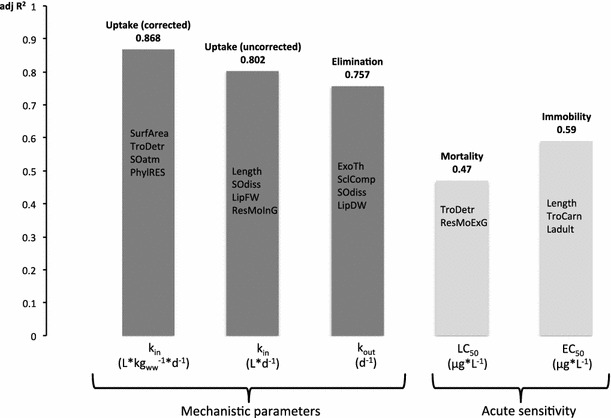
Variance of TK parameters and sensitivity endpoints explained by combinations of traits (adjusted *R* ^2^ of multiple regression analyses). Only most significant trait combinations up to 4 traits per parameter/endpoint are shown and in descending order of significance

The results of the forward multiple regressions are presented in Fig. 2 (most significant relationships) and Supplementary Material C. These will initially be described below in relation to the endpoints, and combinations of up to four traits with high explanatory potential will be indicated. The observed significant trait combinations are numbered in the Supplementary Material C and the text refers to these numbers together with the listing of the relevant traits.

For *k*_in_, eleven significant and two moderately significant trait combinations were found, of which seven were two-way, three were three-way, and another three were four-way combinations. The highest explanatory potential (adj. *R* ^2^ = 0.869) was found for the four-way combination (11) with the traits surface area (SurfArea), TroDetr, SOatm and PhyRES. Also the three-way (8) and the four-way (1) combinations of the same first two or three traits alone explained 83 or 73.1 % of the variance in *k*_in_, respectively. Another combination (9) that explained 72.9 % of the variance in *k*_in_ was composed of the SurfArea, LipFW and SOatm. The lowest amount of variance (R^2^ = 0.494) in *k*_in_ was significantly explained by ResMoCoG and Ljuv. The trait SurfArea appeared the most often in all combinations, followed by SOatm, TroDetr, Ljuv, ResMoCoG, ResMoInG, LipFW, PhyRES, WatCont, BauBox in that order.

For the uncorrected uptake rate constant *k*_in, uncorr_, eleven trait combinations were identified in three two-way, two three-way and six four-way combinations. The four-way combinations explained the highest variation, but were mostly only moderately significant. The highest explanatory but only moderately significant potential was (20)—the four-way combination of SOdiss, LipFW, Length and ResMoInG with an adjusted *R* ^2^ of 0.802. Similarly, although explaining less variance in uptake, but significantly affecting the outcome, it was found that when LipFW was substituted with TroHerb (19), explanatory power increased. Length and SOdiss also explained 74.9–77.7 % variance when combined with ResInt and either BauSphe, TroHerb or PhyRES (22–24). The trait combination ExoTh and SclComp (16) was found to be highly significant, but explained only 39.9 % of the variability in *k*_in, uncorr_. The traits influencing *k*_in, uncorr_ were dominantly Length and SOdiss, followed by BauSphe, LipFW, ResInt, SclComp, ResMoInG, TroHerb, and PhyRES in this order.

Out of the eighteen different trait combinations identified for *k*_out_, thirteen were significantly and five moderately significantly related, explaining between 33.6 and 75.7 % of the variance in elimination rate constants. The highest explanatory power was found for one out of the four trait four-way combinations (42; SOdiss, ExoTh, LipDW, SclComp) and the lowest was found for one out of the eight trait two-way combinations (32; SOdiss, Llarny), while the six three-way combinations showed adjusted *R* ^2^s between 0.534 and 0.71. The two-way trait combination SOdiss and ExoTh (26) explained 46.3 % of the variance in elimination, and each of these two traits appeared in one of the other significant two-way combinations together with other traits (i.e. SclComp, PhyEQ, BauBox ResMoPig and Llarny), but they then explained less variance. The two-way combination explaining the most variance was ResInt with PhyEQ (25, *R* ^2^ = 0.537). The explanatory power of these traits increased when combined with SOdiss (34), although the three-way combination of SOdiss, ExoTh and SclComp (33) explained more variance (*R* ^2^ = 0.65 and 0.71) respectively. The trait LipFW emerged as important in combinations with three and four traits. Clearly, the dominant traits for *k*_out_ were ExoTh and SOdiss followed by SclComp, LipFW, ResInt, Bau Box, PhyEQ, ResMoPig and Llarny in that order.

For BCF_lipid_, eleven different combinations of traits (5 two-way, 3 three-way and 4 four-way combinations) were found with eight being significant and four being moderately significant, explaining between 46 and 79.8 % of the variance in lipid corrected bioconcentration. The most explanatory power was again found for a four-way combination (51; SurfArea, LipFW, TroHerb, PhylEQ) and the least for a two-way combination (47; LipFW, Ladult). SurfArea and LipFW appeared consistently in almost all significant combinations and were (although artefacts of size and lipid correction as discussed above) important if not surprising. Other interesting combinations involve the two-way combination (46) with TroDetr and PhyEQ, explaining 46.7 % variance and the other traits emerging throughout the analyses, which are SclPoor, ResMoCoG, ResInt and Ladult. Dominant traits for BCF_lipid_ were LipFW and SurfArea, followed by PhyEQ, TroDetr, Scl Poor, ResMoCoG, Ladult, ResInt and ExoTh.

For the BCF_ww_, eight combinations of traits were found, of which five were significant and three were moderately significant. Also a four-way trait combination (60; SurfArea, ExoTh, LipDW, SclPoor) explained the most variance in wet weight based bioconcentration (73.6 %), while a two-way combination (56; Ladult, ResMoInG) explained the least (46.9 %). In addition to the SurfArea, Ladult and LipDW also frequently appeared in significant combinations. For instance, the four-way combination (61) with Ladult, LipDW, ResMoInG and WatCont significantly explained 69.7 % of the variance. If the water content in this combination was substituted with TroHerb, somewhat less variance could be explained (64.9 %). The dominant traits for BCF_ww_ were SurfArea, Ladult and LipFW followed by ResMoIng, SclPoor, ExoTh, WatCont, TroHerb and BauSphe in that order.

For the routinely used sensitivity endpoints, both the 48 h L(E)C_50_s together nine combinations of traits were found to be significantly related, while only one two-way combination of traits (63, TroDetr, ResMoExG) significantly correlated with the 48 h LC_50_ (*R* ^2^ = 0.47). The other eight combinations were four two-way or three-way combinations of traits explaining between 30.4 and 59.6 % of the variance in the measured 48 h EC_50_ of which four trait combinations were significant and the other four moderately significant. The most variance in two-way combinations was explained by the combination of Length and TroCarn (adj. *R* ^2^ = 0.41). In addition to these two traits, also Ladult and SclPoor had explanatory power in all possible combinations of these four traits. The highest correlation was detected for the three-way combination Length, Ladult and TroCarn (adj. *R* ^2^ = 0.596) followed by the Length, TroCarn and SclPoor, which explained 53.1 % of the variance, but only at a moderate level of significance. Also the other traits listed for the two-way combinations described above emerge here again in combination with ExoTh and ResMoInG. The traits most dominant in relation to the 48 h EC_50_ were TroCarn followed by Ladult, Length, SclPoor, ExoTh and ResMoInG in that order.

## Discussion

The PCA illustrates that the species selection used was representative of the diversity in traits measured. Similarly, Rubach et al. (2011b) have shown that these same species also represent the range in variation of intrinsic sensitivity to chlorpyrifos in arthropods, which is a prerequisite for the extraction of traits and combinations of traits that may explain differences in intrinsic sensitivity. The PCA furthermore indicates correlations between traits, and these may be due either to the method of quantification (in case of the size-related traits), to structural correlations (for instance the lipid content is naturally correlated to size related measures) or to small phylogenetic distance, leading to ‘trait suites or syndromes’ (Poff et al. 2006; Culp et al. 2011).

The analysis indicates, as would be expected, that size is a strong explanatory and therefore predictive variable for uptake. The negative correlations between elimination and lethality (high 48 h LC_50_ values) lead to the hypothesis that either the elimination rate constant or the internal abilities for repair, recovery and compensation determine survival dynamics. Rubach et al. (2010a) also correlated *k*_out_ in a single linear regression analysis with the 48 h EC_50_ (immobility) and found a significant correlation, (when the species *Neocaridina denticulata* was excluded). Rubach et al. (2011b) discusses the differences in the sensitivity endpoints with immobility and lethality with large focus on delayed mortality in some species, which is the reason for the lack of correlation between these two endpoints in the PCA. This is interesting, as species differences in *k*_out_ could explain differences in sensitivity endpoints (i.e. lethality and immobility), if *k*_out_ is the dominant rate in the process of toxicity.

This exploratory multivariate analysis is informative and provides a good preliminary overview of the link between traits and intrinsic sensitivity, but it is unsuitable for determining significant single traits and combinations of traits that are relevant for intrinsic sensitivity. The results shown in Tables 3 and Supplementary Material B and C show that both single traits and combinations of traits have high explanatory potential for intrinsic sensitivity, as predicted by Baird and Van den Brink (2007) and more specifically for process-related endpoints, such as the toxicokinetic parameters as hypothesized by Rubach et al. (2010a). The amount of variance in endpoints that could be explained using single traits ranged between 0.1 and 56 %, whereas for the selected combinations of traits, much more of the variance (30–87 %, adjusted *R* ^2^) in endpoints was explained (Table 3 and Supplementary Material B and C). In Supplementary Material C the frequency of significant trait occurrences across all analyses is given for each trait, in order to rank their importance as explanatory factor per endpoint. Some traits such as SOdiss, ExoTh, and Ladult become more important overall when analysed in combinations rather than as single traits, and others played a significant role only in combination (TroCarn, Ljuv, TroHerb, and WatCont). These results illustrate that our understanding and therefore ultimately the predictability of intrinsic sensitivity can be enhanced by combining species traits as predictors instead of using only one variable (Rubach et al. 2011a).

Profound differences were seen between the classic sensitivity endpoints and the toxicokinetic endpoints. Firstly, the maximum variance explained by single traits was the highest for the uptake rate constants (56 % by SurfArea) and the lowest for the 48 h EC_50_ (21 % by Ladult), which was similar when combinations of traits were used (86 % for *k*_in_ versus 47 % for the 48 h LC_50_). Secondly, the number of (moderately) significant trait combinations was the highest for *k*_out_ (18 combinations) and the other endpoints related to the toxicokinetics (8–13) in contrast to the EC_50_ (8 combinations) and the LC_50_ (1 combination). Thirdly, the single traits, but also the combinations of traits, found to be significantly correlated with the general toxicity endpoints did not clearly corroborate the understanding of toxicity. Nevertheless, they did confirm the premise that size and life stage are important factors for intrinsic sensitivity and also indicate that detritivore and carnivore trophic relation, poor sclerotization, exoskeleton thickness and respiration mode may also play a role in intrinsic sensitivity.

The correlation of traits with the toxicokinetic parameters establishes a much more mechanistic understanding of how traits influence these processes and therefore contribute to intrinsic sensitivity. For instance, among the size-related traits, organism length explained uptake most successfully. However when uptake was corrected for fresh weight, surface area was a better predictor, indicating that another spatial parameter also plays an important role in these processes and emphasizing adsorption (Table 3). As hypothesized and also supported by previous data (Hendriks 2007; Weiner et al. 2004; Preuss et al. 2008; Rubach et al. 2011a) the AVratio was expected to have the highest explanatory potential for uptake and bioconcentration. Possible explanations of the absence of a direct correlation between AVratio and uptake might be the use of an insufficient quantification technique or too small a difference in AVratio between the selected species.

The positive or negative direction of the relationship between size and uptake and bioconcentration corresponded, as expected, with whether the endpoint variable was corrected (negative for *k*_in_, BCF_ww_, BCF_lipid_) or uncorrected (positive for *k*_in, uncorr_) for fresh weight (Table 3). The BCF_lipid_ was also indirectly corrected for size though the total lipid content. The correlation between LipTot and sensitivity is probably also a size artefact, indicated by correlation coefficients between 0.854 and 0.955 stemming from independent measurements for size related measurements and the total lipid content. The same consideration accounts for the correlations between fresh and dry weight corrected lipid contents and BCF_lipid_, but from the opposite direction and therefore the relationship is negative (Table 3). The traits LipFW and LipDW, which were individually only correlated with the BCF_lipid_, were also found to be important in combinations of three or four traits for uptake, elimination and bioconcentration (Supplementary Material C).

Regression analyses with single traits showed that species with gills are associated with a relatively high uptake of chlorpyrifos, which likely reflects an increased surface area for active or passive uptake. Internal gills, however, correlated with low uptake and bioconcentration (BCF_ww_). Some species with internal branchial chambers such as *A. imperator* close their branchial chambers in order to facilitate ventilation (Mill and Hughes 1966), which if induced by exposure to chemicals (i.e. an avoidance behaviour) may explain this correlation. The source of oxygen for respiration or two modalities for the mode of respiration (ReMoCoG and ResMoInG) enhanced the explanatory potential, when combined with the traits previously mentioned (see combinations 9 and 21). These results agree with those in the literature, as size has been shown to be an important factor for uptake (e.g. Arnot and Gobas 2004; Weiner et al. 2004; Hendriks 2007), as well as lipid content (e.g. Barron 1990; MacKay and Fraser 2000; Hendriks et al. 2005) and respiratory modalities (e.g. Buchwalter et al. 2002, 2003).

Other traits which appeared significant in our analysis such as detritivore or herbivore trophic relation, spherical or box-shaped bauplan, complete sclerotization and high resolution phylogeny may also play an additional role (Supplementary Material C). Since test animals were not fed during the experiments used to parameterize the toxicokinetic model, the correlation between being a detritivore and uptake cannot be related to exposure via food (Table 3), in fact it may be explained by cannibalism, which was observed in the TK experiments, especially for the detritivore and omnivore test species (Rubach et al. 2010a) and is therefore an experimental artefact. Box shape increased both uptake and elimination, which is logical since being dorsoventrally flattened offers a relatively large surface for exchange of chemical. A spherical body has less surface area exposed to the surrounding media relative to the biovolume, and therefore correlated negatively with *k*_in, uncorr_ (*p* ≤ 0.05). This supports the hypothesis that the AVratio plays an important role for uptake and suggests that body shape might be a good and convenient descriptor for this trait. The additional correlation with the 48 h LC_50_ suggests that a thick exoskeleton is related with insensitivity. The relationship with *k*_in, uncorr_, indicated however that concentration increased with increasing exoskeleton thickness, which might be related to sorption of chlorpyrifos to the skeleton itself (Table 3) and be an experimental artefact as, chlorpyrifos, which is locked in the exoskeleton would not be biologically active, but measured by the applied methodology.

For the toxicokinetic process of elimination a very different trait pattern was observed. Size-related traits were not important for this process, neither individually nor in combination. As expected, the analysis showed that a thick exoskeleton and complete sclerotization decreased the speed of elimination. The positive correlation between using dissolved oxygen for respiration (SOdiss) and elimination indicates that respiratory organs are also used for ion exchange, which is broadly known and accepted (e.g. Freire et al. 2008). Larvae or nymphs of insects seemed to be less capable of substance elimination (*p* ≤ 0.05) than other life stages (Table 3). However, this variable was correlated (correlation coefficients between 0.513 and 0.789) with seven other trait modalities (LipDW, ResReg, ResMoPig, SclPoor, SclComp, BauCyl, Ladult) and therefore this should be recognized, but treated with caution. Besides exoskeleton thickness, using dissolved oxygen for respiration and being completely sclerotized, the % lipid of fresh weight (combination 42) was also of importance in addition to several other traits (BauBox, PhyRES, ResMoPig, ResInt and Llarny). Remarkably these traits appeared to be significant in all different combinations and in this case likely interactions with more than four traits might be applicable and appropriate.

Elimination abilities have yet not been subject to many comparative studies and are mostly related to physiological traits investigating detoxification, hence relating enzyme activities with effect responses (Chambers et al. 1994; Chambers and Carr 1995; Printes and Callaghan 2004; Domingues et al. 2010). As no detoxification traits were included in this study (see above), it is difficult to compare the results found to existing knowledge in literature. It is quite likely that such traits would show high explanatory potential if meaningfully quantified (Chambers and Carr 1995; Eaton et al. 2008). The traits that were found to be important in this study might also contribute to elimination, especially because the elimination rates measured were assumed to address the steps after detoxification. It is interesting that phylogeny as a single trait was highly correlated with elimination, because it might indirectly describe well-conserved genes related to detoxification, which are known to exist for drug targets (Gunnarsson et al. 2008). The fact that detoxification and elimination can be appropriately predicted using phylogenetic lineages is also consistent with the findings of Buchwalter et al. (2008), who showed that phylogeny can predict uptake as well as elimination of cadmium in Ephemeroptera, Plecoptera and Trichoptera. However evidence for independent evolutionary invention of enzymes with potential subsequent evolutionally selection has also been described (Galperin et al. 1998). Furthermore, phylogeny will tend to obscure cases where uncommon enzymes, metabolic pathways or modifications cause lower intrinsic sensitivity.

The routinely used ecotoxicity endpoints (LC_50_ and EC_50_) were more difficult to relate to traits or trait combinations directly. The individual traits only confirm size (Length, Ladult) as important, being related to the 48 h EC_50_ and also identify exoskeleton thickness as being related to the 48 h LC_50_. Combinations of traits did not improve the explanation of the LC_50_; only one trait pair was found to be moderately explanatory (TroDetr, ResMoExG). For the 48 h EC_50_, trait combinations explained more of the observed variation. A carnivorous trophic relation, adult life stage, length, poor sclerotization, an internal gill, and exoskeleton thickness appeared to be important explanatory factors in all possible combinations. This result is more informative than the results on individual traits and the results on the 48 h LC_50_, although it still lacks the strong explanatory power of the process-based endpoints. The juvenile trait modality of life stage did not show any significant relationships to any endpoint, which is related to the lack of juvenile data. Only two juvenile life stages were tested and for one species the 48 h L(E)C_50_ of their adult stage was used for the juvenile stage (Table 2). The observed correlation between 48 h L(E)C_50_ with adult life stages is likely to be a size artefact, since adults of the tested species are larger and may have different surface area volume ratios. Other physiological traits, which can be different in juvenile and adult life stages, may also be responsible for differences in the sensitivity observations. In the light of the missing information on the distribution of the biologically active chlorpyrifos oxon throughout the test animals (for which distribution and metabolism both would have to be quantified for each species, also from a trait perspective) this could theoretically become very complex. For a theoretical concept of a model, including also potential physiological traits the reader is referred to Rubach et al. (2011a).

The demonstrated lack of relationship between the routine ecotoxicity sensitivity endpoints and traits is consistent with the findings of Rubach et al. (2010b). They employed an analogous approach to link the largest available existing toxicity dataset, 24–96 h L(E)C_50_ with existing ecological trait information for two mode of actions and three chemical classes. In that study there were no consistent trait patterns using a similar methodological approach. It was therefore concluded that other relevant traits needed to be identified and quantified, and that the processes of toxicity must be considered separately in order to find predictive relationships between traits and intrinsic sensitivity. Similar conclusions can be drawn from this study.

The intrinsic sensitivity of a species is a result of both toxicokinetics and toxicodynamics. Although substantial experimental and theoretical work with a strong emphasis on toxicokinetics and to a lesser extent on toxicodynamics is available in the literature, it is currently unclear whether these processes are of equal importance for determining intrinsic sensitivity. The experiments carried out to parameterize the toxicokinetics of chlorpyrifos in 17 freshwater arthropods showed that a large amount (50–60 %) of the variation in sensitivity (48 h EC_50_) can be explained by the toxicokinetics, i.e. by uptake (32 %) and elimination (28 %) (Rubach et al. 2010a). This estimate is associated with some uncertainty since biotransformation and therefore detoxification was not included. Therefore, toxicokinetics might be considered to have higher explanatory potential. Alternatively, explanatory power might be somewhat overestimated due to adsorption to the outer body parts, which may not necessarily contribute to the concentration at the target site (see above). Despite this uncertainty, these values can be used as an indication for the role of the toxicokinetic processes for intrinsic sensitivity. The remaining variation (40–50 %) might therefore be attributed to biotransformation, distribution of the oxon or the toxicodynamics, namely to the amount of tissue and molecular damage induced, the ability to repair and recover from this damage, and also being able to endure certain internal damage until a threshold is exceeded and effects become visible at the organism level.

This research was performed at the individual-organism level and focused on the determination of intrinsic sensitivity, by posing the question of how relevant such approaches are for ERA. Irrespective of the fact that current ERA practices, at least on the first and second tier, are based on empirical data gathered at the organism level, knowledge and research performed at lower levels of organization than the population, community and ecosystem level are pivotal for the identification of hazard mechanisms, which can be the only basis for truly protective ERA (Van den Brink 2008). In addition, the organism level is the main entity in individual-based population models (IBMs), which offer promising tools for the prediction of population-level dynamics and effects (Galic et al. 2010). Therefore, as we seek to explore and understand such patterns at the organism level, this should, in turn, improve our ability to develop predictive population models. For instance internal concentrations, which significantly determine adverse effects (Meador et al. 2008), cannot be estimated for populations directly without considering phenomena at the organism level. Therefore, knowledge about the intrinsic sensitivity at species level with its underlying processes and factors is important for the future development of holistic and dynamic ecological risk assessment science.

A second reason why predictable intrinsic sensitivity at the species level is important for ERA relates to the fact that, potentially, every chemical can be harmful for some species under certain conditions, but does not necessarily have to be harmful for every species. This means that there are simply too many chemical/species/conditions combinations, which can be practically assessed. If intrinsic sensitivity can be predicted by extrapolating adverse effects at the organism level on basis of species and chemical traits, then the amount of experimental toxicity testing needed and therewith the unnecessary use of test animals could be reduced substantially. In fact, when considering higher organizational levels, such as the community or the ecosystem it is convenient to treat intrinsic sensitivity itself as a trait, which is done in several indices used in biomonitoring, e.g. the SPEAR index (Liess and Von Der Ohe 2005). Therefore, for mechanistic ecotoxicology, prospective ERA, and also retrospective risk assessment (biomonitoring) and approaches which aim at diagnostics of perturbations it would be a major advancement in the field if intrinsic species-related sensitivity could also be predicted.

## Electronic supplementary material

Below is the link to the electronic supplementary material.

Supplementary material 1 (DOC 351 kb)
