# Supplementary material for: Species traits as predictors for intrinsic sensitivity of aquatic invertebrates to the insecticide chlorpyrifos
Source: Ecotoxicology. 2012 Jun 19;21(7):2088–101. doi: 10.1007/s10646-012-0962-8 (PMC3431471; doi:10.1007/s10646-012-0962-8)
Supplement: Supplementary file 1 — Supplementary material 1 (DOC 351 kb) [file 10646_2012_962_MOESM1_ESM.doc]

**Supplemental Material for the paper: *‘Species traits as predictors for intrinsic sensitivity of aquatic invertebrates to the insecticide chlorpyrifos.’***

Mascha N. Rubach^1,2^, Donald J Baird^3^, Marie-Claire Boerwinkel^4^, Stephen J. Maund^2^, Ivo Roessink^4^ and Paul J. Van den Brink^1,4*^

^1^ Department of Aquatic Ecology and Water Quality Management, Wageningen University, P.O. Box 47, 6700 AA Wageningen, The Netherlands.

^2^ Syngenta Crop Protection AG, 4002 Basel, Switzerland

^3^ Environment Canada@Canadian Rivers Institute, Department of Biology, University of New Brunswick, Fredericton, NB, Canada

^4^ Alterra, Wageningen University and Research centre, P.O. Box 47, 6700 AA Wageningen, The Netherlands.

CONTENT

**A:** Traits obtained from literature, databases and through experimental quantification

**B:** Results of single linear regressions

**C:** Results of multiple linear regressions

**D:** References cited in Table 1 (main manuscript)

**A:** Traits obtained from literature, databases and through experimental quantification

*Traits obtained from literature and databases*

Traits that were successfully quantified using existing literature and databases were related to respiration (respiratory regulation (Res), mode of respiration (ResMo) and source of oxygen (SO)), trophic relation (Tro), degree of sclerotization (Scl) and phylogeny (Phy).

For respiration, the literature detailed in Table 1 was reviewed and all relevant information related to respiration was documented. According to Merritt et al. (2008), all aquatic insects, independent of the nature of their respiratory organs, are able to control their oxygen uptake to a certain extent by behavioural patterns such as undulations, swimming abilities, using natural water flow, beating of gills, leg contractions (push-ups), movements and regulating a rectal pump. Therefore, the species were categorized according to their regulatory abilities, following the classification by Merritt et al. (2008) and adding the knowledge gained from the literature and from direct observation in our previous experiments. A species was categorized as a *respiratory regulator* (ResReg), if it had good regulatory abilities (breathing only atmospheric oxygen or breathing dissolved oxygen, but possessing at least two methods to regulate), as an *intermediate respiratory regulator* (ResInt) if one strong method of regulation was found (e.g. rectal pump or being a good swimmer) and *respiratory conformer* (ResConf) when only one (weak) or no method was found for that species (e.g. swimming, but being a bad swimmer). The modalities for the trait 'mode of respiration' described below were also based on Merritt et al. (2008) and characterized at the species level with the literature listed in Table 1. *Cutaneous mode of respiration* (ResMocut) denotes that an animal breathes through the exoskeleton, carapace or a closed tracheal system with apneustic spiracles, and only by diffusion through the cuticle. This occurs in many species as a supplement to gill breathing and could be limited by exoskeleton or cuticular thickness as well as chemical properties (types/layers of oils and waxes) or fugacities (Mackay 2004), which also may change the permeability. We have assumed that cutaneous respiration occurs in all arthropods by default, and this trait would only be meaningful if the respiratory surface area and its permeability were measured precisely. As ResMocut was consequently the same for all species in our study, it was excluded from the quantitative analyses. *Respiration via a siphon* (ResMosip) describes respiration via a tube that connects the atmosphere with an oligopneustic spiracle (open tracheal system) and therefore denotes simple air breathers. Plant breathers pierce macrophytes with modified open tracheal spiracles to tap the plant’s specialized air channels (occurs in syrphids, ephydrids, curculionids and some beetle larvae). As none of the investigated species fell into this category this trait was not listed. *Respiration via a compressible gill* (ResMoCoG) describes a temporary air storage carried along in form of a bubble or plastron (an air film created by hydrofuge hairs). Dissolved oxygen diffuses into this storage, but is consumed quickly and/or the storage is small in relation to the activity levels of the animal, requiring frequent surfacing to replenish atmospheric oxygen (open tracheal system, mostly with polypneustic spiracles). Similarly, an incompressible gill is also a plastron or a spiracle with which the animal can dive, but no reloading with atmospheric oxygen is needed. Hydrofuge hairs or other structures create a permanent air storage in the form of a gaseous film where dissolved oxygen constantly diffuses at the rate proportional to oxygen consumption (open tracheal systems with poly- or oligopneustic spiracles, common in some Coleoptera, Hemiptera and the lepidoteran family Pyraloidea and some families of Diptera). It was not clear whether the species *Parapoynx stratiotata*, a pyraloid moth, makes respiratory use of a plastron (Thorpe 1950) in addition to functional tracheal external gills (Welch 1922). Therefore this trait modality was not included in the database. *Respiration through external gills* (ResMoExG) describes very thin-walled and highly tracheated appendices and outgrowths connected to apneustic spiracles (closed tracheal system). External gills significantly increase the permeable surface area available for diffusion through the cuticle. The functionality of external gills for oxygen uptake is a subject of great debate in many species and these features are generally assumed to be more important for ion exchange and the creation of currents to enhance respiration through the body surface area. *Respiration via internal gills* (ResMoInG) denotes functional gills situated in internal rectal or lateral branchial chambers. Some insect taxa (Notonectidae: Anisops and Buenoa, Chironomidae) and most crustaceans exhibit *respiratory pigments* (ResMoPig), such as haemoglobine and haemocyanine (Crustacea) in their haemolymphe, thereby increasing the oxygen transport. Maltby (1995) found that *Asellus aquaticus* is five times less sensitive to hypoxia than *Gammarus pulex,* and related this finding to differences in blood characteristics. In this study, all Crustaceans were categorized as using respiratory pigments because no detailed information was available on species level. More simply (but perhaps more meaningfully for bioconcentration than the previous categorizations), is the differentiation of species in relation to their use of *atmospheric or dissolved source of oxygen*. In order to categorize species into atmospheric (SOatm) and dissolved oxygen (SOdiss) breathers, the information collated above was used.

For quantification of feeding-related traits, no distinction was made between the various modes of feeding (e.g. scraping, collecting, filtering, piercing, engulfing), because all but one test species fell into the category of collectors. However, a distinction was made between preferred food types, here called the trophic relation. Of the species studied, 90% of the species were observed to ingest algae, biofilms and detritus attached to preferred food items (Merritt et al. 2008). This 'passive omnivory' is not used further here but categorizations are made on the basis of an organisms' active food selection: *detritivore* (TroDetr), *herbivore* (TroHerb), *carnivore* (TroCarn) and omnivore (TroOmni) *trophic relation*. To categorize the species, we used the literature reported in Table 1 and also our own experience from the experimental work reported in Rubach et al. (2010a; 2011b).

The degree of body sclerotization was assessed by using the trait ‘armouring’ from the USGS database (Poff et al. 2006) supplemented with photographs collected in this study for the measurement of morphometric traits (see below). A *poor degree of sclerotization* was noted if sclerites were ≤ 10% or non-existent (SclPoor), a *good degree of sclerotization* was scored if coverage was between 10 to 90 % (SclGood), and *complete sclerotization* was indicated if 90 to 100% of the body surface was sclerotized or the species was equipped with a carapace (SclComp).

The trait 'bauplan' was quantified with close consideration of the trait surface area/ volume ratio (AVratio) described below. For the calculation of the latter, geometric shapes were assumed for each species, and the shapes were used to categorize the bauplan. The categories used were: *box shape bauplan* (BauBox) for rectangular shaped species that appear to be dorso-ventrally or laterally flattened; *cylindrical shape bauplan* (BauCyl) included all circular and elliptic-cylindrical formed shapes; *spherical shape bauplan* (BauSphe) included ball and ellipsoid-like species; and *cone shape bauplan* (BauCone) was used for fully conic and dorso-ventrally-flattened conic or semi-conic organisms.

The life stage of each species investigated was categorized as *adult* (Ladult), *larval or nymph* (Llarny) or *juvenile* (Ljuv) *life stage*, based on the life-stage used in experiments described previously.

In order to address the evolutionary descent of the investigated species, their relative phylogenetic position was estimated. For this the Tree of Life web project (Maddison and Maddison 1996) was used (retrieved from <http://tolweb.org> on the 01.09.2009) to construct a phylogenetic tree to the highest taxonomic resolution possible for each species, which were Anisoptera, Asellota, Chaoboridae, Ephemeroptera, Culicidae, Cladocera, Amphipoda, Molannidae, Caridea, Notonectidae, Crambidae, Pleidae, Cambaridae, Nepidae and Sialidae. For the *highest resolution phylogeny* (PhyRES), the number of nodes to the common arthropod ancestor was counted back from these taxa. For the *equalized phylogeny* (PhyEQ) the number of nodes from the order level equivalents for each species was counted back to the common arthropod ancestor. For both quantifications, the number of nodes was transformed into ranks: 1 (1 node, oldest) to 11 (23 nodes, youngest) for PhyRES and ranks 1 (1 node, oldest) to 7 (12 nodes, youngest) for PhyEQ. It has to be noted that this quantification of phylogeny does not entirely describe how similar the taxa are, because the phylogenetic ‘between taxa distances’ were not accounted for.

All of the traits addressed above, except the phylogenetic quantifications, were binary coded, meaning that the database entry was 0, if a trait modality was non-existent in a species and 1 if it was existent, while it was possible to be assigned a 1 for multiple trait modalities of one trait. PhyRES and PhyEQ were ordinal variables, which corresponded to the rankings described above.

*Experimentally quantified metric traits*

The traits experimentally quantified in this study were all body-size related traits (biovolume, length, surface area, AVratio, dry weight), water content, lipid content and exoskeleton thickness. For lipid content, sub-samples from the toxicokinetic experiments were mostly used, and the methodology and detailed results for this trait are reported and discussed in Rubach et al. (2010a). For the current study, lipid content was expressed as *total lipid content* (LipTot), *% lipid in fresh weight* (LipFW) and *% lipid in dry weight* (LipDW). For most of the other measurements, animals were collected from the field as reported in SM Table 1 (date, origin), together with information on several methodological details. In order to investigate representative samples with respect to the toxicity, toxicokinetic and toxicodynamic studies described in Rubach et al. (2010a; 2011b) or due to a lack of presence, some species (denoted by the condition ‘dead’ in SM Table 1) traits were measured using surplus or sub-samples of previous samples stored at -20°C.

For the quantification of *surface area* (SurfArea), *biovolume* (Biovol), *surface area/volume ratio* (AVratio) and *length* (Length), species-specific distance measures were taken according to previously assigned geometric bodies and their respective formulas for surface area and biovolume, for which the length was always a necessary measure. This approach has been previously applied for phytoplankton (Hillebrand et al. 1999), but has been considered difficult to apply to aquatic invertebrates (Magnusson et al. 2003). SM Table 1 informs on the geometric bodies assigned to the species and Figure 1 shows the used equations and formulas for the geometrical bodies.

Some species were assigned only one geometric form, but for others combinations (summations and subtractions of different geometrical bodies) were used. For instance, the surface area of *Daphnia magna* was described using the surface area of an ellipsoid body in addition to the surface area of an ellipse, the latter of which stands for the folded inner part of the carapace, which is exposed to the outer medium (Pirow et al. 1999b, a). In general, all appendices, such as antennae, legs or siphons were not included in the measures. To obtain the required measures for the larger sized animals, a ruler or millimetre paper was used, while the smaller animals were placed under a stereo microscope (Olympus SZX 10) and digital pictures of the specimen were taken in dorsal and lateral views using a digital camera (Axiocam ICc3, Carl Zeiss, The Netherlands). Subsequently, to extract the required distance measures, the pictures taken were analysed with the AxioVision 4.8 software (Carl Zeiss, The Netherlands), which was previously calibrated to the magnifications used for each photograph. The calculations for the size-related traits were made individually for the measured specimen and the average value for each species was incorporated into the trait database.

The determination of dry weight and water content was conducted in accordance with Wetzel et al. (2005) by placing individual specimen into clean previously weighed aluminium boats, reweighing these and drying at constant 103°C for at least 24 h to a constant dry weight. All weights were determined on a microbalance and from the loss measures, the dry weight and water content were calculated. Specimen of *Anax imperator*, *Cloeon dipterum*, *Molanna angustata*, *Notonecta maculata*, *Parapoynx stratiotata* and *Ranatra linearis* were defrosted and blotted dry before measurement. Due to loss of samples, the values in the database for the water content of *Culex pipiens* and *D. magna* originate from the parallel dry weight determination for lipid content, as described in Rubach et al. (2010a; 2011b). All individually calculated dry weights and water contents were averaged and these values are listed in the trait database.

Exoskeleton thickness was determined using frozen organisms from the samplings listed in SM Table 1 and again for the species *R. linearis*, *N. maculata*, *A. imperator* and *P. stratiotata* from subsamples of the toxicokinetic studies Rubach et al. (2010a). Animals were cut into several pieces in such a way that the animals were opened appropriately to ensure tissue digestion and also to ensure that representative pieces of the animal were collected. For instance, in case of *R. linearis* a leg, a claw, part of the siphon, a cross cut piece of the abdomen, the thorax, the head and the wings were included into the vial for subsequent tissue digestion. The tissue digestion was carried out with different volumes of tissue solubiliser (Soluene-350, Perkin Elmer Inc.). The amount of Soluene-350 varied between 1.5 mL and 10 mL, depending on the amount of tissue and the time needed (varied between 24 h and 11 d) to fully digest the tissue in a water bath of 60°C. The remains of this digestion process were washed thoroughly with ethanol to remove the Soluene-350 and any tissue leftovers. Afterwards the exoskeletons were stored in ethanol until microscopic analysis. Before measurement of the thickness of the randomly collected parts of the exoskeleton, these were cut into cross sections which were then placed on a glass slide under a stereomicroscope. For some species, auxiliary means were used to stabilize the sectional planes. Also, exoskeletons of some species were easier to measure when the ethanol had evaporated and others needed the suspension in either water or ethanol. The following measurement of the exoskeleton thickness was conducted using the same set-up with the stereomicroscope, camera and associated software, as described for the size related traits above. The individual measures taken were averaged and included as such into the trait database.

**Results and Discussion**

The trait database is displayed in SM Table 2 and relates to the information compiled in Table 1. This database is a combination of experimentally-quantified traits and literature information and generally contains wholly new information not present in other existing trait databases. The matrix consists of 12 different traits, expressed as 37 trait modalities (and/ or different ways of quantification) in total, which were scored for 17 ‘species’ (15 taxonomic species of which two were quantified separately for juvenile and adult stages). The information in the database is coded in three different ways: metric, binary and ordinal. Previous trait databases have been either binary (Heneghan et al. 1999) or fuzzy coded (Chevenet et al. 1994; Usseglio-Polatera et al. 2000b), with the latter approach providing a higher resolution when assigning trait modalities to species, because one species can have more than one trait modality, expressed as ‘affinities’ to a certain trait modality. The application of this approach facilitated the inclusion of trait modalities which were present in all species investigated (i.e. inclusion of ResMocut or omnivory). Nevertheless in the present study, the binary approach was preferred in order to reduce complexity, and this was required as the combined datasets were each expected to introduce high background variability.

Although, on the one hand, the database is not yet comprehensive, especially with regard to physiological traits (see discussion below), it already contains a large number of trait modalities compared to the number of species included. Hence the variance in the explanatory variables (traits) could exceed the variance in the response variables (sensitivity) when used in further analyses. For this reason, the number of traits was reduced systematically before using the database. For example, only the most relevant size-related traits were included with regard to each response variable in question. Nevertheless the database should in future be extended, since the missing physiological traits are of major importance with regard to intrinsic sensitivity, and are therefore important to be meaningfully quantified. Traits which were originally intended to be quantified but could not be addressed in the present study included the *complexity of the nervous system*, the *metabolic rate*, the *amount of target receptors* (acetylcholinesterase), and the *detoxification abilities* of a species. We were unable to quantify these traits from the literature because most work on these traits has been performed only for certain model species and hardly any comparative studies on freshwater arthropods exist to date. The other option, namely to quantify these type of traits experimentally was tempting, because of the expected strong relationships to the toxicodynamics and the possibly improved interpretation of the toxicokinetic patters observed by Rubach et al. (2010a; 2011b). Although this was beyond the scope of this thesis, these traits are discussed further below.

In order to measure the *complexity of the nervous system* several approaches were considered. The general neuro-anatomy of insects consists of the central nervous system with brain, ventral nerve cord and ventral ganglia plus the peripheral nerve system. Interesting ways of approaching its complexity could have been to determine the number of different cell types in the nervous system, or extracting the complete central nervous system, counting the number of ganglia and fused ganglia and calculating their ratio. The more fused ganglia an insect has, the more complex the nervous system might be, and thereby the organism’s nervous system might be either easier disrupted by neurotoxic compounds or have better compensatory abilities. The other option would be to base phylogeny on the nervous system (Harzsch 2006) and assume that complexity increases in evolution (Valentine et al. 1994; McShea 1996, 2000; McCarthy and Enquist 2005).

The variety of methods available to quantify the *amount of target receptors* was manifold. A relatively straightforward approach would have been to measure enzyme activities of acetylcholinesterase as a biomarker in the test species under control conditions. It was unclear however, how comparable the data would be across species, since the used test animals were sampled from different sites/cultures, with different background history, at different times/ seasons and under different conditions (Domingues et al. 2010). Therefore we would always only obtain ‘snapshots’ of enzyme activity, which would not necessarily be specific to the species. From the body of literature reviewed, it was concluded that in order to interpret such biomarker data in a comparative approach, extensive experimental work would have to be performed. In more detail, to be able to relate this information systematically to differences in species sensitivity, it would have even been necessary to investigate differences in enzyme constitution, their affinities to chlorpyrifos in addition to concentration and time dependent differences in transcription rates and gene expression of the acetylcholinesterase in all species. Similar difficulties were encountered for the quantification of the detoxification abilities, however, here the situation turned out to be even more complex, because although many detoxification mechanisms are known, more enzymes and also the feedback loops of different stress response systems are involved (Korsloot et al. 2004). Feedback loops were also hypothesized to play a role for the quantification of target receptors due to receptor aging and *de novo* synthesis. In the future, research such as Heckmann et al. (2008) has conducted for *D. magna*, will enable comparative studies, because modern techniques such as next generation sequencing genomes of species will be rapidly and easily available. Also, other techniques such as DNA bar-coding (Valentini et al. 2009) will contribute to revisiting phylogeny and support the genetic description of traits (see also below).

**SM Table 1:** Methodological details on the experimentally measured traits.

| **Species** | **Sampled** | **Origin** | **Life stage** | **Measured** | ***n* (size)** | ***n* (dry weight)** | ***n***  **(ExoTh)** | **Size method** | **Condition** | **Geometric formula** | **TK sub-sample** | **TK match** |
| --- | --- | --- | --- | --- | --- | --- | --- | --- | --- | --- | --- | --- |
| *Anax imperator* | 06.11.2006 | SH ditches | Larva ^a^ | 5/8.7.2007 | 37 | 37 | 11 | Ruler | dead | elliptic cylinder | no | very good |
| *Asellus aquaticus* | 15.06.2009 | SH cosms | Adult | 15.06.2009 | 20 | 20 | 10 | Camera / software | alive | rectangular box | no | fair |
| *Chaoborus obscuripes* | 10.03.2009 | SH cosms | Larva | 10.03.2009 | 20 | 19 | 4 | Camera / software | alive | circular cylinder | no | very good |
| *Cloeon dipterum* ^b^ | 14.05.2009 | SH cosms, ditches | Larva | 14.05.2009 | 10 | 25 | 3 | Camera / software | alive | rectangular box | no | very good |
| *Culex pipiens* | 09.6.2009 | SH tank | Larva | 09.6.2009 | 9 | 20 | 4 | Camera / software | alive | circular cylinder+prolate sphere+sphere | no | very good |
| *Daphnia magna* | 01.05.2009 | Culture | Adult | 01.05.2009 | 20 | 19 | 5 | Camera / software | alive | ellipsoid + elliptic A | no | very good |
| *Gammarus pulex* (AD) ^c^ | 22.06.2009 | SH storage basin | Adult | 22.06.2009 | 20 | 20 | 5 | Camera / software | alive | 2 circular cylinders subtracted | no | very good |
| *Gammarus pulex* (JU) ^c^ | 16.06.2009 | SH storage basin | Juvenile | 16.06.2009 | 20 | 20 | 6 | Camera / software | alive | 2 circular cylinders subtracted | no | very good |
| *Molanna angustata* | 03.07.2009 | Groene Heuvel | Larva | 03.07.2009 | 20 | 3 | 3 | Camera / software | alive | elliptic cylinder | no | very good |
| *Neocaridinia denticulata* ^c^ | 31.03.2009 | Culture | Adult | 31.03.2009 | 20 | 20 | 12 | Camera / software | alive | rectangular box | no | very good |
| *Notonecta maculata* | 06.08.2007 | SH cosms | Adult | 23.06.2009 | 17 | 3 | 11 | Camera / software | dead | cone | yes | very good |
| *Paraponyx stratiotata* ^d^ | 06.08.2007 | SH cosms | Larva | 09.07.2009 | 20 | 3 | 6 | Camera / software | dead | elliptic cylinder | yes | very good |
| *Plea minutissima* | 25.03.2009 | SH cosms | Adult | 25.03.2009 | 20 | 20 | 16 | Camera / software | alive | elliposid | no | fair |
| *Procambarus spec.* (AD) | 08.06.2009 | Culture | Adult | 08.06.2009 | 40 | 20 | 6 | Ruler | alive | elliptic cylinder | no | very good |
| *Procambarus spec.* (JU) | 08.06.2009 | Culture | Juvenile | 09.06.2009 | 20 | 20 | 11 | Camera / software | alive | elliptic cylinder | no | very good |
| *Ranatra linearis* | 06.11.2006 | SH ditch 20 | Adult | 5/8.7.2007 | 38 | 38 | 5 | Ruler | dead | rectangular box | no | very good |
| *Sialis lutaria* | 22.06.2009 | SH ditch 16 | Larva | 23.06.2009 | 14 | 18 | 11 | Camera / software | alive | half cone + triangle | no | very good |

^a^ Larvae stage three

^b^ 20 specimens measured (10 solely in top view and the other 10 solely in lateral view, measures merged to 3D measures of 10 animals)

^c^ Length measured in fragments for bend lines, (upper bend and lower bend measured separately, sum of fragments averaged)

^d^ Length measured in fragments for bend lines

*Note:*

‘*n*’ denotes the sample size. ‘ExoTh’ refers to the trait ‘Exoskeleton thickness’.

‘SH’ denotes the experimental field station of Alterra, ‘The Sinderhoeve', Renkum, The Netherlands.

‘TK’ refers to the toxicokinetic experiments (Rubach et al. 2010a; 2011b)

‘Groene Heuvel' is a sandy oligotrophic lake in Wijchen, The Netherlands.

The equations for the geometrical bodies used are given in Figure 1.

Alive animals were anaesthetized for measurements.

**SM Table 2:** The trait by species database, see Table 1 for the explanation of the short-form names and the units of the traits.

*Note:*

*D. magna* /ResMoCut: respiration is enhanced through the inner shell and the rostrum area.

*A. aquaticus*, *G. pulex*, *N. denticulata*, *Procambarus* spec. /ResMoExG: the external gills are probably covered with a cuticule, which shed with mould.

*C. dipterum* /ResMoExG: unclear if external gill is functional.

*S. lutaria* /ResMoExG*:* external gills are much more permeable than the rest of the body surface.

*P. stratiotata*/ ResMoExG: possibility that the external gills are dysfunctional and respiration occurs via a plastron (incompressible gill).

AD denotes adult specimen, JU juvenile specimen

**
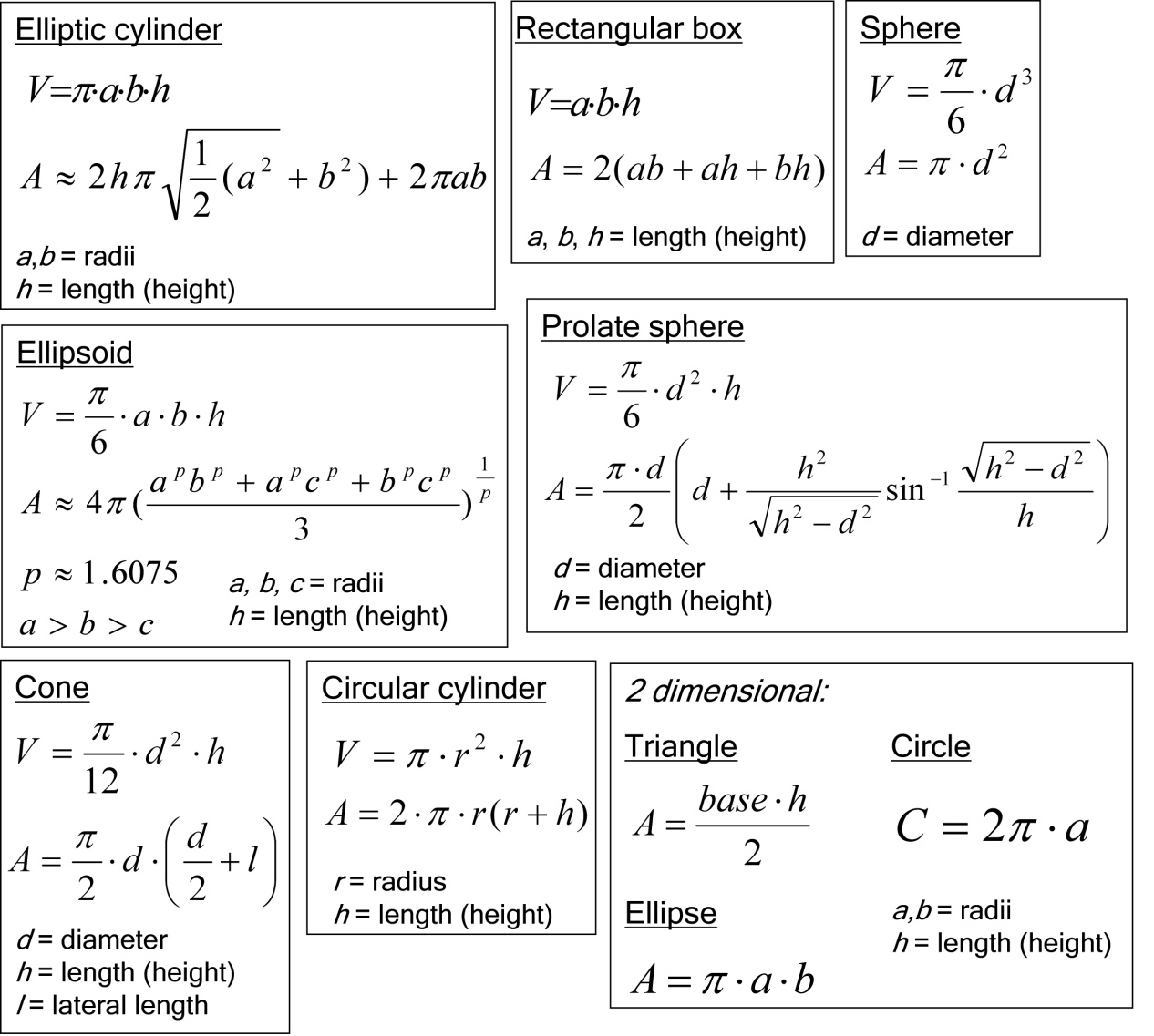
**

**SM Figure 1:** Equations and formulas for geometrical bodies used to quantify the size related traits for the different species. Formulas and approach were taken from Hillebrand et al. (1999), Wikipedia at [http://en.wikipedia.org/wiki/Ellipsoid retrieved on 14.08.2009](http://en.wikipedia.org/wiki/Ellipsoid%20retrieved%20on%2014.08.2009) (surface area of ellipsoid) and efunda at <http://www.efunda.com/math/solids/IndexSolid.cfm> (retrieved on 13.08.2009). *V* = (bio)volume); *A* = surface area; *C* = circumference. An organism’s length mostly corresponded either to *h* (height) or a specific diameter, while the width and thickness were mostly diameters. MS Table 1 shows which combinations of geometrical bodies were used for each species.

**B: Detailed results of single linear regressions**

| **Trait (modality)** | ***k_in_* [L**·**kg_ww_^-1^** ·**d^-1^]** | | ***k_in_uncorr* [L**·**d^-1^]** | | ***k_out_* [d^-1^]** | | **BCF_ww_ [L**·**kg_ww_^-1^]** | | **BCF_lipid_ [L**·**kg_lipid_^-1^]** | | **48h LC_50_ [µg**·**L^-1^]** | | **48h EC_50_ [µg**·**L^-1^]** | |
| --- | --- | --- | --- | --- | --- | --- | --- | --- | --- | --- | --- | --- | --- | --- |
|  | ***P*** | **adj. R^2^** | ***p*** | **adj. R^2^** | ***p*** | **adj. R^2^** | ***p*** | **adj. R^2^** | ***p*** | **adj. R^2^** | ***p*** | **adj. R^2^** | ***p*** | **adj. R^2^** |
| Biovol | 0.001** | 0.471 | 0.005** | 0.379 | 0.714 | NC | 0.011** | 0.316 | 0.05** | 0.182 | 0.842 | NC | 0.117 | 0.1 |
| SurfArea | **< 0.001**** | **0.56** | 0.01** | 0.326 | 0.727 | NC | **0.004**** | **0.391** | **0.041**** | **0.2** | 0.991 | NC | 0.144 | 0.079 |
| AVratio | 0.87 | NC | 0.682 | NC | 0.674 | NC | 0.656 | NC | 0.725 | NC | **0.11** | **0.106** | 0.291 | 0.012 |
| Length | 0.001** | 0.474 | **< 0.001**** | **0.545** | 0.646 | NC | 0.014** | 0.298 | 0.074* | 0.143 | 0.527 | NC | **0.045**** | **0.191** |
| DryMass | 0.005** | 0.384 | 0.008** | 0.338 | **0.59** | **NC** | 0.036** | 0.213 | 0.09* | 0.125 | 0.752 | NC | 0.13 | 0.089 |
| WatCont | **0.895** | **NC** | **0.788** | **NC** | **0.627** | **NC** | **0.643** | **NC** | **0.856** | **NC** | **0.326** | **0.002** | **0.751** | **NC** |
| ExoTh | **0.102** | **0.113** | **0.055*** | **0.173** | **0.042**** | **0.197** | **0.871** | **NC** | **0.603** | **NC** | **0.009**** | **0.329** | **0.325** | **0.002** |
| LipFW | **0.297** | **0.01** | **0.317** | **0.004** | 0.893 | NC | 0.379 | NC | **0.006**** | **0.37** | 0.645 | NC | 0.743 | NC |
| LipDW | 0.428 | NC | 0.598 | NC | **0.834** | **NC** | **0.373** | **NC** | 0.066* | 0.156 | **0.119** | **0.098** | 0.824 | NC |
| LipTot ^a^ | < 0.001** | 0.558 | 0.004** | 0.397 | 0.734 | NC | 0.004** | 0.392 | 0.012** | 0.31 | 0.717 | NC | **0.134** | **0.086** |
| ResConf | 0.346 | NC | 0.935 | NC | 0.557 | NC | 0.642 | NC | 0.801 | NC | 0.318 | 0.004 | **0.318** | **0.004** |
| ResInt | **0.077*** | **0.14** | **0.253** | **0.025** | **0.272** | **0.018** | **0.401** | **NC** | **0.263** | **0.022** | 0.756 | NC | 0.882 | NC |
| ResReg | 0.454 | NC | 0.256 | 0.021 | 0.626 | NC | 0.716 | NC | 0.427 | NC | **0.253** | **0.025** | 0.481 | NC |
| SOatm | **0.017**** | **0.278** | 0.284 | 0.015 | 0.113 | 0.103 | **0.321** | **0.003** | 0.383 | NC | 0.454 | NC | **0.524** | **NC** |
| SOdiss | 0.493 | NC | **0.23** | **0.034** | **0.064*** | **0.157** | 0.562 | NC | **0.27** | **0.019** | **0.338** | **NC** | 0.737 | NC |
| ResMocut | NP | NP | NP | NP | NP | NP | NP | NP | NP | NP | NP | NP | NP | NP |
| ResMosip | 0.493 | NC | 0.23 | 0.034 | 0.064 | 0.157 | 0.562 | NC | 0.27 | 0.019 | 0.228 | NC | 0.737 | NC |
| ResMoCoG | 0.206 | 0.044 | 0.099* | 0.116 | 0.938 | NC | 0.263 | 0.022 | **0.059*** | **0.166** | 0.973 | NC | 0.572 | NC |
| ResMoExG | 0.027** | 0.237 | 0.162 | 0.068 | 0.253 | 0.025 | 0.25 | 0.026 | 0.3014 | 0.008 | **0.206** | **0.044** | 0.827 | NC |
| ResMoInG | **0.013**** | **0.304** | **0.064*** | **0.158** | 0.897 | NC | **0.029**** | **0.231** | 0.105 | 0.11 | 0.718 | NC | **0.485** | **NC** |
| ResMoPig | 0.187 | 0.054 | 0.198 | 0.048 | **0.023**** | **0.252** | 0.811 | NC | 0.427 | NC | 0.642 | NC | 0.532 | NC |
| TroDetr | **0.086*** | **0.129** | 0.878 | NC | **0.202** | **0.046** | 0.495 | NC | **0.168** | **0.064** | **0.131** | **0.089** | 0.767 | NC |
| TroHerb | 0.323 | 0.003 | **0.743** | NC | 0.854 | NC | **0.426** | **NC** | 0.541 | NC | 0.546 | NC | 0.534 | NC |
| TroCarn | 0.175 | 0.06 | 0.79 | NC | 0.274 | 0.018 | 0.623 | NC | 0.968 | NC | 0.803 | NC | **0.128** | **0.091** |
| TroOmni | 0.525 | NC | 0.852 | **NC** | 0.507 | NC | 0.897 | NC | 0.623 | NC | 0.5 | NC | 0.505 | NC |
| SclPoor | **0.25** | **0.026** | 0.378 | NC | 0.27 | 0.019 | **0.055*** | **0.172** | **0.337** | **NC** | 0.766 | NC | **0.126** | **0.092** |
| SclGood | 0.283 | 0.015 | 0.196 | 0.05 | 0.146 | 0.078 | 0.983 | NC | 0.391 | NC | **0.209** | **0.043** | 0.738 | NC |
| SclComp | 0.824 | NC | **0.063*** | **0.16** | **0.025**** | **0.244** | 0.087* | 0.128 | 0.83 | NC | 0.504 | NC | 0.264 | 0.02 |
| BauBox | **0.087*** | **0.128** | 0.78 | Nc | **0.073*** | **0.145** | 0.725 | NC | 0.435 | NC | 0.343 | NC | **0.478** | **NC** |
| BauCyl | 0.362 | NC | 0.348 | NC | 0.203 | 0.046 | 0.986 | NC | 0.996 | NC | 0.88 | NC | 0.591 | NC |
| BauSphe | 0.76 | NC | **0.029**** | **0.232** | 0.335 | NC | **0.34** | **NC** | **0.274** | **0.018** | 0.997 | NC | 0.561 | NC |
| BauCone | 0.458 | NC | 0.849 | NC | 0.1 | 0.115 | 0.694 | NC | 0.946 | NC | **0.089*** | **0.12** | 0.723 | NC |
| Ladult | 0.441 | NC | **0.69** | **NC** | 0.159 | 0.07 | **0.081*** | **0.135** | **0.215** | **0.041** | 0.825 | NC | **0.038**** | **0.206** |
| Llarny | 0.886 | NC | 0.863 | NC | **0.048**** | **0.186** | 0.142 | 0.081 | 0.573 | NC | 0.618 | NC | 0.147 | 0.077 |
| Ljuv | **0.328** | **0.001** | 0.723 | NC | 0.468 | NC | 0.686 | NC | 0.302 | 0.009 | **0.261** | **0.022** | 0.392 | NC |
| PhylRES | **0.217** | **0.039** | **0.425** | **NC** | 0.043** | 0.194 | 0.859 | NC | 0.61 | NC | **0.456** | **NC** | 0.7 | NC |
| PhylEQ | 0.671 | NC | 0.485 | NC | **0.014**** | **0.295** | **0.245** | **0.028** | **0.541** | **NC** | 0.588 | NC | **0.647** | **NC** |

**C:** Results of multiple linear regressions (F statistic of combinations of trait modalities for each sensitivity endpoint,)

^a^ no combinations with *p* ≤ 0.1found

^b^ only combinations with *p* ≤ 0.05 shown, all analysed subsets were significant.

*Note:*

*- p* value reports the F statistic, notation for significance: * = *p* ≤ 0.1 and ** = *p* ≤ 0.05.

- adj. R^2^ = adjusted coefficient of determination, percent variance accounted for by trait combination.

- Never in a significant combination: Biovol, AVratio, Dry Mass, ResConf, ResReg, ResMoSip, TroOmni, SclGood, BauCyl, BauCone.

- LipTot and ResMocut were not included in analyses (see text for explanation).

- Grey shaded areas depict the most significant combination for each sensitivity endpoint.

**D:** References cited in Table 1

Babula A (1979) Ultrastructure of respiratory epithelium in fresh-water isopod Asellus aquaticus L. (Crustacea). Acta Medica Polona 20:355-356

Brown SD (1960) The ingestion and digestion of algae by Cloeon dipterum L. (Ephemeroptera). Hydrobiologia 16:81-96

Freire CA, Onken H, McNamara JC (2008) A structure-function analysis of ion transport in crustacean gills and excretory organs. Comp Biochem Physiol, A 151:272-304

Gupta TS, Stewart KW (2000) Life history and case building behavior of Molanna tryphena (Trichoptera: Molannidae) in two East Texas spring-fed streams. Ann Entomol Soc Am 93.

Hickin NE (1967) Caddis larvae - larvae of the British Trichoptera. Hutchinson and Co, Ltd., London, UK.

Jalihal DR, Almelkar GB, Sankolli KN (1994) Atyid Shrimps of the Genus Caridina H. Milne Edwards, 1837. Potential Crustacean Material for Experimental Biology. Crustaceana 66:178-183

Locklin JL, Arsuffi TL, Bowles DE (2006) Life history of Sialis (Megaloptera: Sialidae) in a lentic and lotic ecosystem in Central Texas. Am Midl Nat 155:50-62

Maddison DR, Maddison WP (1996) The Tree of Life Project. URL http://tolweb.org/tree/

Maltby L (1995) Sensitivity of the Crustaceans Gammarus pulex (L.) and Asellus aquaticus (L.) to short-term exposure to hypoxia and unionized ammonia: Observations and possible mechanisms. Water Res 29:781-787

McShaffrey D, McCafferty WP (1990) Feeding behavior and related functional morphology of the mayfly Ephemerella needhami (Ephemeroptera: Ephemerellidae). J Insect Behav 3:673-688

Merritt RW, Cummins KW, Berg MB (2008) An introduction to the aquatic insects of North America. 4th edition. Kendall Hunt Publishing, Dubuque, Iowa, USA.

Mill PJ, Hughes GM (1966) The nervous control of ventilation in dragonfly larvae. J Exp Biol 44, 297-316.

Pirow R, Wollinger F, Paul RJ (1999) The sites of respiratory gas exchange in the planktonic crustacean Daphnia magna: An in vivo study employing blood haemoglobin as an internal oxygen probe. J Exp Biol 202:3089-3099

Poff NL, Olden JD, Vieira NKM, Finn DS, Simmons MP, Kondratieff BC (2006) Functional trait niches of North American lotic insects: Traits-based ecological applications in light of phylogenetic relationships. J North Am Benthol Soc 25:730-755

Rubach MN, Ashauer R., Maund SJ, Baird DJ, Van den Brink PJ (2010a) An experimental study of toxicokinetic variation in 15 freshwater arthropod species exposed to the insecticide chlorpyrifos. Environ Toxicol Chem 29:2225-2234

Schuh RT, Slater JA (1995) True bugs of the world (Hemiptera: Heteroptera) - classification and natural history. Cornell University Press, Ithaka, NY, USA.

Steele DH, Steele VJ (1991) Effects of salinity on the survival, growth rate, and reproductive output of Gammarus lawrencianus (Crustacea, Amphipoda). Mar Ecol Prog Ser 78:49-56

Taylor HH, Taylor EW (1992) Gills and lungs: the exchange of gases and ions. In: Microscopic anatomy of invertebrates: Decapod Crustaceans (Eds. Harrison FW, Humes AG). Wiley Liss, Inc., New York, USA, pp. 203-293

Ueno M, Inoue Y, Niwa N (1997) Podocytes of the freshwater shrimp - Fine structure and effect of injected trypan blue. J Electron Microsc 46:485-490

Welch PS (1922) The respiratory mechanism in certain aquatic Lepidoptera. T Am Microsc Soc 41:29-50

Wichard W (1978) Structure and function of the tracheal gills of Molanna angustata. In: 2nd International Symposium on Trichoptera (ed. Crichton MI, Junk W) The Hague, The Netherlands, pp. 293 - 296

Williams WD (1962a) The genus Asellus in Britain. Nature 193:900-901

Williams WD (1962b) Notes on the ecological similarities of Asellus aquaticus (L.) and A. meridianus Rac. (Crust., Isopoda). Hydrobiologia 20:1-30

Wingfield CA (1939) Function of the gills of mayfly nymphs from different habitats. J Exp Biol 16:363-373

Yee DA, Kesavaraju B, Juliano SA (2004) Larval feeding behavior of three co-occurring species of container mosquitoes. J Vect Ecol 29:315-322
